# Hypopigmentation following intradermal allergy skin testing performed as part of mRNA COVID-19 vaccine allergy evaluation

**DOI:** 10.5339/qmj.2022.fqac.9

**Published:** 2022-04-01

**Authors:** Asaad Imameldin, Yaldez Ibrahim, Tayseer Ibrahim, Hassan Mobayed

**Affiliations:** ^1^Adult Allergy and Immunology Division, Department of Medicine, Hamad Medical Corporation, Doha, Qatar E-mail: AImameldin@hamad.qa

**Keywords:** COVID-19 vaccine allergy, hypopigmentation, IDT

## Abstract

Background: Skin prick test (SPT) and intradermal test (IDT) are standard procedures in the allergy practice that are safe when performed. Individuals with a history of allergic reaction to the COVID-19 vaccine can undergo allergy skin testing for polyethylene glycol and polysorbate 80 to determine their eligibility for the same vaccine or a safe alternative.

Hypopigmentation is an infrequent adverse effect of corticosteroids, including triamcinolone acetonide, following local and intralesional treatment. Exposure to high potency corticosteroids for a long duration and the intradermal injection route are risk factors for hypopigmentation.

In this case report, we describe the development of hypopigmentation following triamcinolone ID testing.

Case report: A 29-year-old lady with a history of immediate severe allergic reaction following the first dose of mRNA COVID-19 vaccine (Pfizer) underwent SPT and IDT for polysorbate 80 and polyethylene glycol. Triamcinolone acetonide and Prevnar 13 were used as an indicator of polysorbate 80. Following a negative SPT, IDT for triamcinolone acetonide was negative at 1:10 of 40 mg/mL and positive at 1:1 of 40 mg/mL. A few days later, she noticed hypopigmented lesions at the site of the intradermal skin test for both concentrations of triamcinolone. The lesions have increased in size since then (see image). The patient was diagnosed with steroid-induced hypopigmentation secondary to triamcinolone IDT injection.

Conclusion: Skin hypopigmentation following intraarticular and intralesional triamcinolone injection has been reported previously. However, to the best of our knowledge, this is the first reported case of steroid-induced hypopigmentation following intradermal skin testing. Furthermore, this report highlights that even a low dose of local triamcinolone can cause hypopigmentation. We believe that this case report regarding the rare adverse event will alert clinicians to the potential complication of corticosteroid IDT and help them counsel the patients and provide a thorough explanation before any procedure.

## Figures and Tables

**Figure 1.**
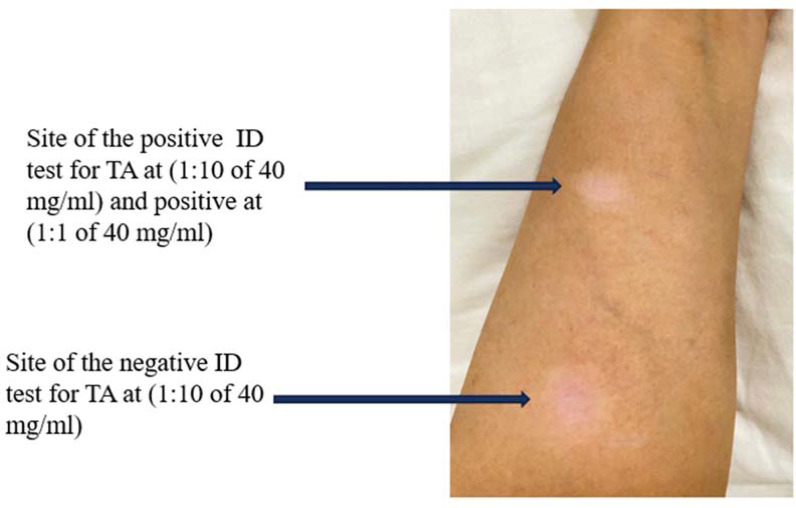
1): Site of the intradermal test and hypopigmentation at the volar surface of the left arm. Intradermal. TA: triamcinolone acetonide.

